# Enhanced Recovery After Surgery Guidelines and Hospital Length of Stay, Readmission, Complications, and Mortality: A Meta-Analysis of Randomized Clinical Trials

**DOI:** 10.1001/jamanetworkopen.2024.17310

**Published:** 2024-06-18

**Authors:** Khara M. Sauro, Christine Smith, Seremi Ibadin, Abigail Thomas, Heather Ganshorn, Linda Bakunda, Bishnu Bajgain, Steven P. Bisch, Gregg Nelson

**Affiliations:** 1 Department of Community Health Sciences and O’Brien Institute of Public Health, Cumming School of Medicine, University of Calgary, Calgary, Alberta, Canada; 2 Department of Surgery, Cumming School of Medicine, University of Calgary, Calgary, Alberta, Canada; 3 Department of Oncology and Charbonneau Cancer Institute, Cumming School of Medicine, University of Calgary, Calgary, Alberta, Canada; 4 Libraries and Cultural Resources, University of Calgary, Calgary, Alberta, Canada; 5 Department of Obstetrics and Gynecology, Cumming School of Medicine, University of Calgary, Calgary, Alberta, Canada; 6 Ariadne Labs, Brigham and Women’s Hospital, Harvard T.H. Chan School of Public Health, Boston, Massachusetts

## Abstract

**Importance:**

A comprehensive review of the evidence exploring the outcomes of enhanced recovery after surgery (ERAS) guidelines has not been completed.

**Objective:**

To evaluate if ERAS guidelines are associated with improved hospital length of stay, hospital readmission, complications, and mortality compared with usual surgical care, and to understand differences in estimates based on study and patient factors.

**Data Sources:**

MEDLINE, Embase, Cumulative Index to Nursing and Allied Health Literature, and Cochrane Central were searched from inception until June 2021.

**Study Selection:**

Titles, abstracts, and full-text articles were screened by 2 independent reviewers. Eligible studies were randomized clinical trials that examined ERAS-guided surgery compared with a control group and reported on at least 1 of the outcomes.

**Data Extraction and Synthesis:**

Data were abstracted in duplicate using a standardized data abstraction form. The study followed the Preferred Reporting Items for Systematic Reviews and Meta-Analyses. Risk of bias was assessed in duplicate using the Cochrane Risk of Bias tool. Random-effects meta-analysis was used to pool estimates for each outcome, and meta-regression identified sources of heterogeneity within each outcome.

**Main Outcome and Measures:**

The primary outcomes were hospital length of stay, hospital readmission within 30 days of index discharge, 30-day postoperative complications, and 30-day postoperative mortality.

**Results:**

Of the 12 047 references identified, 1493 full texts were screened for eligibility, 495 were included in the systematic review, and 74 RCTs with 9076 participants were included in the meta-analysis. Included studies presented data from 21 countries and 9 ERAS-guided surgical procedures with 15 (20.3%) having a low risk of bias. The mean (SD) Reporting on ERAS Compliance, Outcomes, and Elements Research checklist score was 13.5 (2.3). Hospital length of stay decreased by 1.88 days (95% CI, 0.95-2.81 days; *I^2^* = 86.5%; *P* < .001) and the risk of complications decreased (risk ratio, 0.71; 95% CI, 0.59-0.87; *I^2^* = 78.6%; *P* < .001) in the ERAS group. Risk of readmission and mortality were not significant.

**Conclusions and Relevance:**

In this meta-analysis, ERAS guidelines were associated with decreased hospital length of stay and complications. Future studies should aim to improve implementation of ERAS and increase the reach of the guidelines.

## Key Points

**Question:**

Does the adoption of enhanced recovery after surgery (ERAS) guidelines decrease hospital length of stay, hospital readmission, complications, and mortality?

**Findings:**

In this meta-analysis of 74 randomized clinical trials with 9076 participants, the ERAS guidelines were associated with decreased hospital length of stay and complications. Type of surgery and number of ERAS elements were associated with estimates of length of stay.

**Meaning:**

These findings suggest that the ERAS guidelines were associated with decreasing hospital length of stay and complications; future research should focus on improving implementation and compliance with ERAS guidelines to improve patient outcomes.

## Teaser

This meta-analysis assesses the association of the enhanced recovery after surgery guidelines and hospital length of stay, readmission, complications, and mortality compared with usual care.

## Introduction

Globally, more than 313 million people undergo lifesaving or life-prolonging surgery yearly and that number is increasing.^1,2^ While surgery is essential to maintain health and quality of life, it consumes significant health care resources and is not without complications. The mean length of hospital stay for surgery is 5 days equating to more than 7.5 million patient days each year at a cost of at least $1200 per day.^1^ The length of hospital stay increases for patients undergoing surgery who experience complications postoperatively.^3,4,5,6,7,8,9,10^ More than a third of patients undergoing surgery experience complications,^11,12,13^ placing these patients at higher risk of complications than patients not undergoing surgery.^3,4,5,14,15^ Patients who experience complications are also more likely to require additional interventions, be readmitted to hospital, and die, which are all associated with increased costs.^10,16,17,18^ An estimated 50% of complications are preventable, which provides an opportunity to use evidence-based strategies to improve outcomes for patients and health care systems.^3,4,8,9,10,11,13,14^ Moreover, improving surgical outcomes and reducing health care resources can increase the capacity for patients to access surgical care.^8,9^

As the need for surgery continues to exceed health care capacity, strategies are needed to ensure care is effective, efficient, and safe. One option is implementing the enhanced recovery after surgery (ERAS) guidelines. There are currently ERAS guidelines for 23 types of surgery^19^ and implementation programs active in more than 20 other types of surgery.^20^ ERAS guidelines provide recommendations for perioperative care and have been found to reduce complications,^21^ length of hospital stay,^22^ and cost.^23,24^ Several surgery-specific systematic reviews have found positive or mixed effects on outcomes after adopting ERAS guidelines.^25,26,27,28,29,30,31^ However, a comprehensive review of ERAS efficacy across surgical areas and within heterogenous populations has not been conducted. To our knowledge, there are no systematic reviews examining these outcomes across the breadth of ERAS-guided surgical procedures or comparing outcomes by surgery type, which could provide a better understanding of ERAS efficacy. The objective of this study is to synthesize the evidence on the efficacy of ERAS and determine if the adoption of ERAS guidelines improves hospital length of stay, hospital readmission, mortality, and postoperative complications compared with usual surgical care?

## Methods

This systematic review and meta-analysis is reported using the Preferred Reporting Items for Systematic Reviews and Meta-analyses (PRISMA) reporting guideline.^32^ The protocol was registered with the PROSPERO registry of systematic reviews (CRD42021255973).

### Search Strategy

The search strategy combined controlled vocabulary and keywords for the population (*patients undergoing surgery*), the intervention (*ERAS*, *fast track*, *enhanced recovery*) and outcomes (*length of hospital stay*, *hospital readmission*, *complications*, *mortality*) and was run in MEDLINE, Embase, the Cumulative Index to Nursing and Allied Health Literature, and Cochrane Central Register of Controlled Trials on June 18, 2021 (eAppendix 1 in Supplement 1). The search strategy was not limited by publication date or language.

### Eligibility Criteria

Studies were included if they reported at least 1 of the outcomes among adult patients undergoing surgery before and after ERAS guidelines were implemented. Studies that implemented ERAS guidelines, including ERAS-based guidelines (eg, ERAS, fast-track, enhanced recovery protocol), were included if the protocols contained elements that were ERAS-based. Any study design that compared the intervention (ERAS guidelines) and the comparator group (usual care) were included in the overall systematic review, but only randomized clinical trials were included in the meta-analysis.

Studies with only pediatric patients or those that did not stratify estimates by adults and children were excluded. Studies that did not provide estimates or sufficient data to calculate estimates were excluded. Conference proceedings or abstracts, reviews, guidelines, and commentaries were excluded. Studies with less than 50 patients total were excluded due to the high risk of bias in these studies. Studies published in any language were included; however, it was excluded if we could not translate the article. For studies with multiple manuscripts, the manuscript with the most comprehensive dataset was included and the others were excluded (duplicate data).

### Study Selection

Covidence was used to manage study selection.^33^ At each stage of screening a sample of titles and abstracts (n = 5), and full texts (n = 10) were screened by all reviewers to ensure consistency between reviewers and additional studies were reviewed together until a κ of 0.8 was achieved. Titles, abstracts, and full texts were screened for eligibility by 2 independent reviewers. During the title and abstract screening, references included by at least 1 reviewer were included for full-text screening to decrease the risk of incorrectly excluding studies. Disagreement between reviewers was resolved through discussion or consulting a third reviewer.

### Data Collection Process

A standardized database (Microsoft Excel) was used to abstract data from included studies. The data abstraction database was pilot tested on 2 studies. Prior to full data abstraction all reviewers were trained by completing data abstraction for the same 5 studies; training continued until a κ of 0.8 was achieved. Data were abstracted by a single reviewer and 65% of the data were double-checked by an independent reviewer. The data dictionary for the standardized data abstraction form is available in eAppendix 2 in Supplement 1.

### Variables

The primary outcome variables were hospital length of stay, readmission within 30 days of index hospital discharge, postoperative complications within 30 days of surgery, and mortality within 30 days of surgery. While the primary outcome variables were limited to 30 days from surgery or index hospitalization, data for other time periods were abstracted as secondary outcomes.

Additional data items abstracted from the studies included bibliographic information, study characteristics, patient data, type of protocol evaluated (ERAS, enhanced recovery, fast-track, other), number of ERAS elements included in the protocol (count) even if compliance was not reported for each element, and data on compliance. A full list of data items abstracted is included in the data dictionary (eAppendix 2 in Supplement 1).

### Risk of Bias in Individual Studies

The risk of bias of individual studies was evaluated using the Cochrane Risk of Bias Tool (version 2),^34^ by 2 independent reviewers with graduate training in epidemiology. Any disagreements between reviewers were resolved through discussion. The Reporting on ERAS Compliance, Outcomes, and Elements Research (RECOvER) checklist assessed completeness of reporting.^35^

### Data Analysis

Study characteristics were described using descriptive statistics as appropriate. A random effects meta-analysis was used to pool estimates for each outcome variable (metan package).^36,37^ The *I^2^* quantified the magnitude of between-study heterogeneity, and the Cochrane Q statistic determined the significance of heterogeneity. Risk ratios (RR) were calculated for data reported as proportions (ie, mortality, complications, and readmission), and mean differences were calculated for data reported as means (ie, hospital length of stay). Estimates reported as median (IQR) were converted to mean (SD) using the Wan method^38^ and were pooled.

Meta-regression explored potential sources of heterogeneity for each outcome. The potential sources of heterogeneity included patient age and sex, year(s) of data collection, type of surgery, number of ERAS elements included in the protocol, type of protocol (eg, ERAS, enhanced recovery protocol, fast-track, or other), and RECOvER scores. Publication bias was assessed through visual inspection of a funnel plot and statistically using Egger linear regression method. All analyses were performed in Stata 20.0 (StataCorp).^36,37^ Data were analyzed from September 2023 to February 2024. Statistical tests were 2-sided, and significance was set at *P* < .05.

## Results

The search strategy yielded 12 047 unique references, of which 1493 were reviewed in full-text, 495 were included in the overall systematic review (eFigure in Supplement 1) and 74 RCTs ^39,40,41,42,43,44,45,46,47,48,49,50,51,52,53,54,55,56,57,58,59,60,61,62,63,64,65,66,67,68,69,70,71,72,73,74,75,76,77,78,79,80,81,82,83,84,85,86,87,88,89,90,91,92,93,94,95,96,97,98,99,100,101,102,103,104,105,106,107,108,109,110,111,112^ were included in the meta-analysis. The characteristics of each individual study are included in eTable 1 in Supplement 1. The included studies represent 9076 patients with 4577 patients in the control (non-ERAS) group and 4375 patients in the ERAS group. In the 54 studies that included age, the mean (SD) age was 50.0 (12.6) years for patients in the control group and 49.2 (12.6) years among patients in the ERAS group.^39,41,42,43,44,45,46,47,49,50,51,52,53,54,56,57,59,62,63,64,65,66,67,68,70,73,76,79,81,82,83,84,86,88,89,91,93,94,95,96,97,98,99,100,101,102,103,104,105,106,108,109,110,111^

### Study Characteristics

The median (range) year of publication was 2019 (2009-2021). Most studies were conducted in high income and high-middle income countries (57 [80.3%]); 22 (29.7%) were published in China,^40,46,55,56,67,68,69,70,71,72,75,82,85,86,87,103,107,108,109,112,113^ 8 (10.8%) in India,^44,45,60,76,84,91,97,100^ 5 (6.8%) in the US.^47,49,66,99,104^

**Figure 1.**
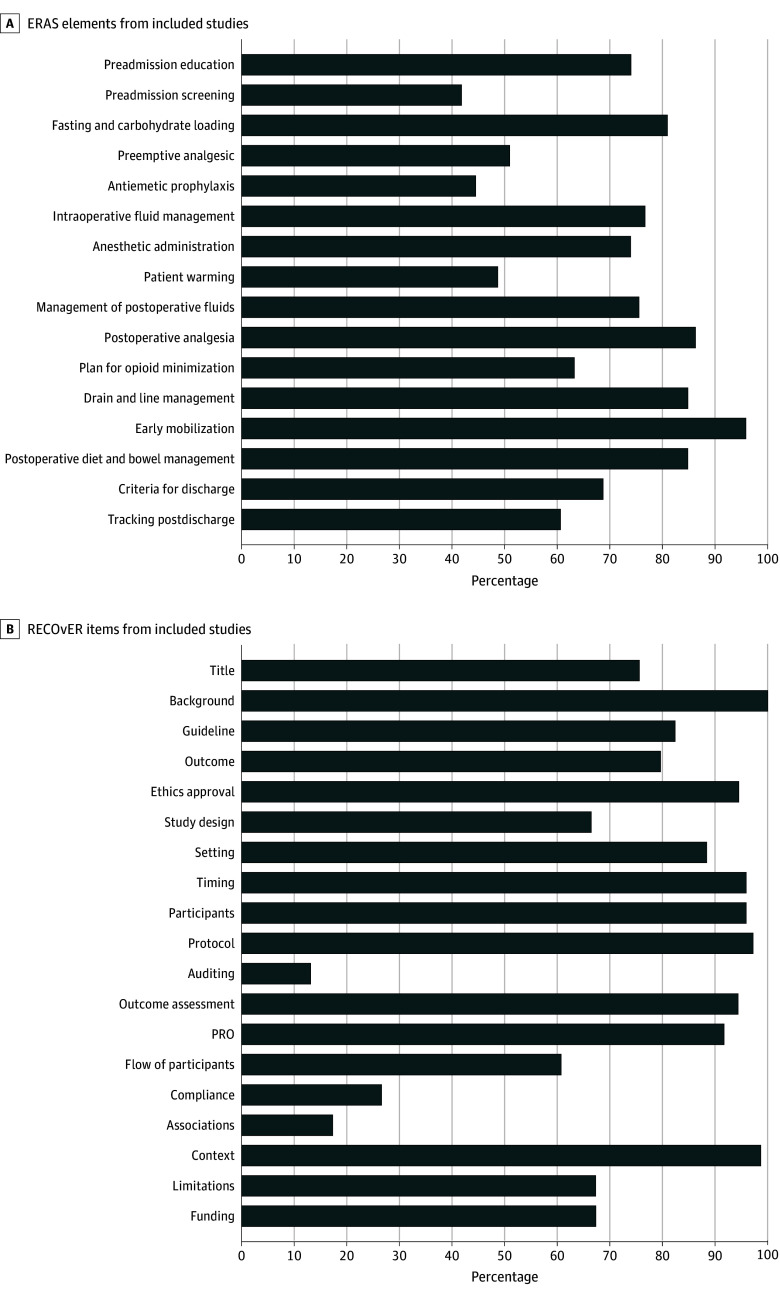
Study Characteristics (A) Among the studies that reported the enhanced recovery after surgery (ERAS) elements, most included early mobilization, postoperative analgesia considerations, and postoperative diet and bowel management. (B) Reporting on ERAS Compliance, Outcomes, and Elements Research (RECOvER) checklist items of included studies. PRO indicates patient reported outcomes.

Most of the 74 studies examined the outcomes of ERAS in patients undergoing gastrointestinal surgery (32 [43.2%])^39,40,45,47,50,51,53,54,56,58,59,60,73,74,77,79,80,83,87,92,93,94,95,96,97,98,100,102,103,105,112,114^ and gynecological surgical procedures (11 [14.9%]),^41,43,49,52,65,89,90,110,111,115^ with only 1 (1.4%) in cardiac surgery,^68^ and 2 (2.7%) in urology.^44,106^ Most of the 74 studies evaluated the outcomes of ERAS guidelines (37 [50.0%]),^39,40,41,42,43,44,45,46,48,52,53,54,57,68,69,70,71,73,75,78,82,87,88,89,90,91,98,99,100,101,104,105,106,107,108,110,111^ whereas 23 (31.1%) evaluated enhanced recovery protocols,^47,49,55,56,58,61,62,63,64,65,66,76,77,79,80,81,83,84,85,95,96,97,102^ 13 (17.6%) evaluated fast-track protocols,^50,51,59,60,72,74,86,92,93,94,103,109,112,116^ and 1 (1.4%) evaluated other types of protocols or pathways based on ERAS principles.^67^ The mean (SD) number of ERAS elements included in the interventions (ERAS, enhanced protocols, fast-track, other) was 11.1 (3.0) elements of a possible 16. Commonly early mobilization (71 [96.0%]), postoperative analgesia management (64 [86.5%]), postoperative diet and bowel management (63 [85.1%]), and drain and tube management (63 [85.1%]) were included (Figure 1). Protocols classified as ERAS included more elements than those classified as fast-track but were no different than enhanced recovery protocols (*F*_1,493_ = 9.58; *P* = .002). The mean (SD) number of elements among studies of ERAS was 11.8 (2.8), compared with 8.9 (2.3) in fast-track protocols, 10.6 (3.7) in enhanced recovery protocols, and 11.4 (3.2) elements with other types of protocols. Compliance with ERAS protocols was reported in 17 (20.3%) studies^49,52,53,56,59,67,69,70,77,80,88,89,90,99,101,106,107^ but was inconsistently reported and could not be pooled; the mean (SD) compliance reported was 74.7% (10.2%) from the 9 studies from which we could synthesize the data.^49,52,67,80,89,90,99,101,106^

### Meta-Analysis by Outcome

#### Length of Stay

Of the 74 included studies, 44 studies (62.2%) reported an overall median or mean estimate of hospital length of stay.^39,40,42,44,45,47,48,49,50,52,53,55,58,60,61,65,66,71,73,74,78,79,82,84,85,88,89,90,91,95,97,98,101,103,105,107,108,109,111,113^ The pooled mean length of hospital stay was 1.88 days (95% CI, 0.95-2.81 days; *I^2^* = 86.5%; *P* < .001) shorter in the ERAS groups compared with control groups. ERAS also decreased the postoperative hospital length of stay (22 studies; mean difference, 2.83 days; 95% CI, 2.10-3.55 days; *I^2^*  = 0%; *P* < .001) compared with control groups. Publication bias was not significant.^40,41,53,54,56,63,64,68,71,72,75,79,82,85,87,94,104,105,107,112,113,117^

#### Hospital Readmission

Nineteen of 74 studies (41.3%) reported 30-day hospital readmissions. The pooled RR of 30-day hospital readmission was 1.04 (95% CI, 0.81-1.35; *I^2^* = 0%; *P* = .74)^39,43,44,52,53,54,65,66,67,69,79,80,89,94,97,101,102,105,112^; with a protective effect when the time period was not specified (11 studies, RR, 0.61; 95% CI, 0.41-0.93; *I^2^* = 0%; *P* = .02).^41,42,47,56,63,75,82,88,98,106,111^ Publication bias was not significant.

#### Complications

Fourteen of 74 studies (18.9%) reported an overall (unstratified) estimate of complications with a pooled estimate of 0.71 (95% CI, 0.59-0.87; *I^2^* = 78.6%; *P* < .001),^44,46,53,67,68,69,79,80,82,86,87,93,95,96^ favoring ERAS compared with control groups. Of the nine studies that reported 30-day complications, the pooled RR was 0.73 (95% CI, 0.56-0.94; *I^2^* = 86.3%; *P* < .02),^53,67,79,80,82,87,93,95,96^ favoring ERAS over control groups. There was evidence of publication bias using the Egger test for small study effects (*t* = 2.24; 95% CI, 0.14-2.15; *P* = .003); however, estimates were symmetrically distributed upon visual inspection of the funnel plot.

#### Mortality

There were 19 of 74 studies that reported 30-day postoperative mortality, with a pooled risk of 0.95 (95% CI, 0.48-1.88) with no heterogeneity between estimates (*I^2^ *= 0%; *P* = .89).^40,44,50,53,54,57,69,70,77,78,87,89,92,93,95,96,97,98,107^ Because many studies reported estimates of 0 mortality, a continuity correction was applied to calculate RRs.^118^ Publication bias was not significant.

### Sources of Heterogeneity Determined by Meta-Regression

**Figure 2.**
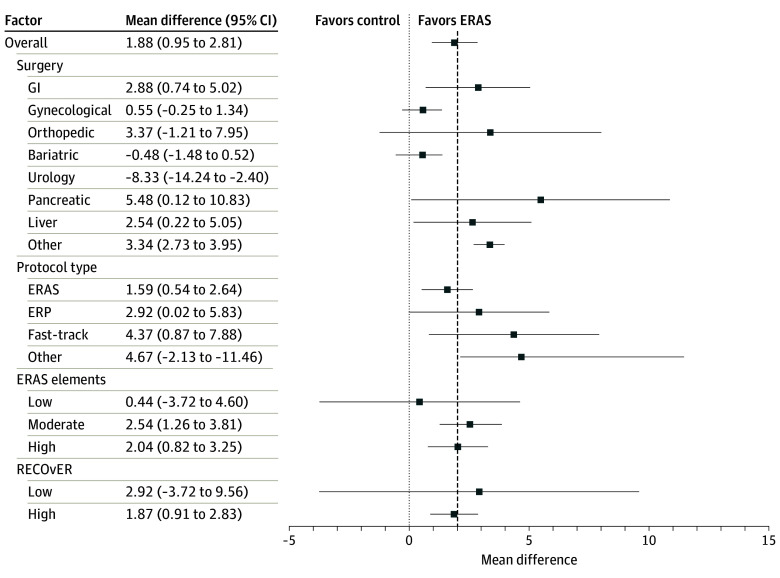
Meta-Analysis of Hospital Length of Stay Patients in enhanced recovery after surgery (ERAS) groups stayed 1.9 days less in hospital compared with control groups. Patient undergoing pancreatic, orthopedic, and gastrointestinal (GI) surgical procedures had greater reductions in hospitals stays than patients undergoing gynecological and breast surgery. ERP, enhanced recovery protocol; RECOvER, Reporting on ERAS Compliance, Outcomes, and Elements Research.

**Figure 3.**
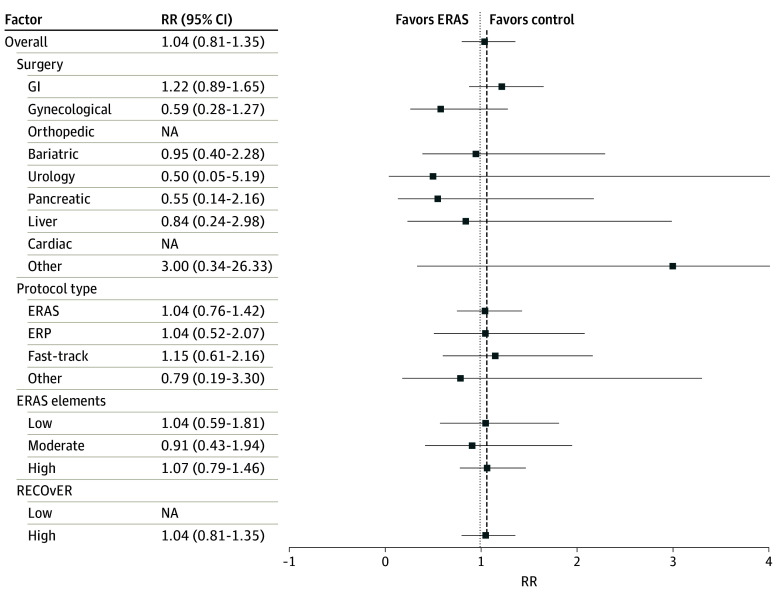
Meta-Analysis of Hospital Readmission Patients in the enhanced recovery after surgery (ERAS) groups had a lower risk of being readmitted after their index hospitalization than patients in the control group. None of the variables explored were associated with the risk of readmission. ERP indicates enhanced recovery protocol; GI, gastrointestinal; NA, not applicable; RECOvER, Reporting on ERAS Compliance, Outcomes, and Elements Research; RR, risk ratio.

**Figure 4.**
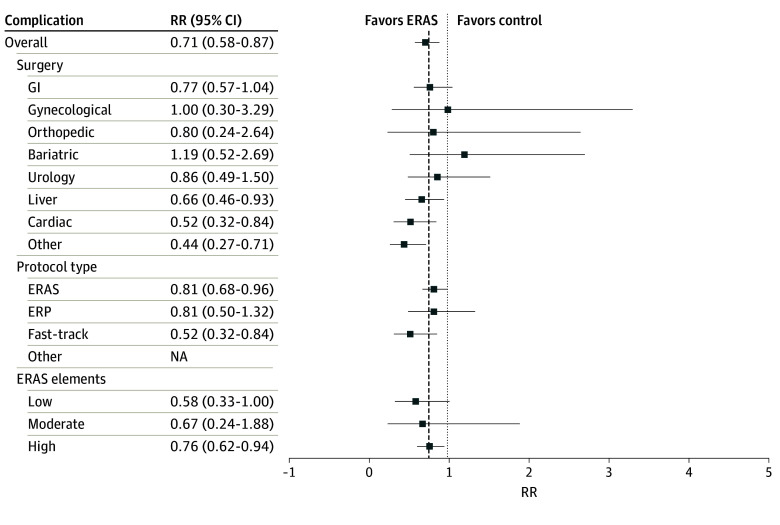
Meta-Analysis of Complications Patients in the enhanced recovery after surgery (ERAS) groups had a lower risk of having complications after their surgery than patients in the control group. None of the variables explored were associated with the risk of complications. ERP indicates enhanced recovery protocol; GI, gastrointestinal; NA, not applicable; RR, risk ratio.

There were no differences in the pooled estimates of hospital length of stay based on country income, RECOvER checklist score, year of publication, number of ERAS elements included in the protocol, or risk of bias. The type of surgery accounted for heterogeneity in the estimate of hospital length of stay (coefficient, 0.32; 95% CI, 0.17-0.48; *P* < .001). The pooled estimate of mean difference in length of hospital stays for patients undergoing pancreatic, orthopedic, and gastrointestinal surgery was larger than other surgical procedures (Figure 2). None of the variables explored accounted for variance in the pooled estimate of 30-day hospital readmission, complications, or mortality. These findings can be found in Figure 3 and Figure 4.

### Risk of Bias and RECOvER

Among the 74 included studies, 40 (54.1%) had some risk of bias, 19 (25.7%) had high risk of bias, and 15 (20.3%) had low risk of bias. Missingness of outcome data were considered a source of low risk of bias among 64 studies (86.5%) while deviations from the per-protocol intervention was considered a source of high risk of bias in 17 studies (23.0%) (eTable 2 in Supplement 1).

The mean (SD) number of RECOvER checklist items that were included in the studies was 13.5 (2.3). Commonly reported items included providing context and importance within the background (74 [100.0%]), explaining the significance of the study (73 [98.7%]), and reporting the protocol used (72 [97.3%]) (Figure 1). The least commonly reported items were auditing of outcomes (10 [13.5%]), examining associations between outcomes and other variables (13 [17.6%]), and reporting compliance with the ERAS guidelines (20 [27.0%] (Figure 1).

## Discussion

This study found that using the ERAS guidelines were associated with a decrease in the length of hospital stay and complications. Hospital length of stay varied by surgery type. There were many studies included, but some evidence gaps remain. For example, there was a paucity of evidence for some surgical procedures (none for head and neck and breast, and few studies for cardiac), fewer studies reported mortality, and compliance was reported inconsistently and poorly.

While the ERAS guidelines were established with the publication of the ERAS Society guideline for perioperative care of patients undergoing colonic surgery,^119^ enhanced recovery as a concept was based on previously described fast-track protocols.^120,121,122^ Accordingly, it is not surprising that many included studies were labeled as fast-track and that a large proportion were focused on gastrointestinal surgery. However, this study did not identify any differences in estimates of hospital length of stay, readmission, complications, or mortality related to the protocol name. While the name given to the protocol examined did not account for heterogeneity between estimates, the number of ERAS elements included in the protocols did for length of hospital stay. This suggests that the number of elements included in the protocol is likely more important than the branding of the protocol, which is supported by previous studies.^123,124^ Encouragingly, most studies reported their protocol elements (90%), which is important for evaluating ERAS outcomes,^35^ but very few studies reported compliance with each element. Unfortunately, most studies included less than 70% (11) of the traditional ERAS elements. While the number of ERAS elements included in the protocol is important, it was not possible to assess what elements are most important for the outcomes of ERAS. Future studies should focus on evaluating the relative outcomes of each ERAS element to understand the core elements required to achieve the benefits of ERAS. This will simplify ERAS and promote adoption in lower-resource settings.^125,126^

Like with other guidelines, ERAS outcomes depends on adoption of ERAS guidelines.^127,128,129^ Indeed, greater compliance with ERAS has been shown to improve outcomes.^129,130,131^ Since only about 20% of included studies reported compliance with ERAS, and reporting of these estimates were too heterogeneous to include in the analysis, it is likely that mean compliance was lower than 75% and that our findings underestimate the outcomes of ERAS. If compliance is important for ERAS outcomes, how can compliance be improved? Strategies for implementing ERAS have been outlined, with a focus on team building and audit of compliance-and-feedback.^126,132^ These strategies are aligned with principles of implementation science, and evidence-informed strategies for effective guideline implementation.^127,128^ Additional strategies that should be considered when implementing ERAS include identifying barriers and facilitators to implementation, including end-users in developing strategies, education, and prompts-and-reminders.^127,133,134^ Taken together, future work should focus on strategies to improve implementation of and compliance with ERAS guidelines; the importance of which has been noted.^126^ Additionally, in agreement with the RECOvER checklist, we advocate for improved reporting of overall compliance and compliance with each ERAS element in studies exploring the outcomes of ERAS guidelines.

### Limitations

This study has limitations. While the search strategy was developed by a librarian with expertise in systematic reviews, some studies may not have been identified. Using the Egger statistic and a visual assessment of funnel plots, we did not find evidence of bias toward studies with large effect sizes. While the comprehensiveness of the systematic review is a strength, it can also be limiting because included studies were heterogeneous based on the type of ERAS protocol, surgery type, and outcomes. Additionally, analysis is limited by the data provided within the included studies. As such, variables that may be associated with our outcome variables, including socioeconomic status, insurance status, and surgical complexity, were not accounted for in the analysis. Similarly, patients undergoing ERAS-guided surgical procedures may be a highly selected group, which we could not control for. Finally, these findings may not be generalized to all types of surgery because most studies explored the efficacy of ERAS in colorectal surgery and obstetric or gynecologic surgery. Indeed our findings suggested that the type of surgery was associated with length of hospital stay, but the reason for the variation was unclear.

## Conclusions

This meta-analysis found that ERAS was associated with a decreased length of hospital stay (without a significant increase in hospital readmissions) and complications. Given the improvements across specialties, future research should aim to apply ERAS to new surgical specialties and in more clinical settings globally.

## Notes

Supplement 1. **eAppendix 1.** Search Strategy **eAppendix 2.** Data Dictionary **eTable 1.** Characteristics of Included Studies **eTable 2.** Risk of Bias **eFigure.** PRISMA Flow Diagram

Supplement 2.
Data Sharing Statement
