# Errors in Figures

**DOI:** 10.1001/jamanetworkopen.2024.28433

**Published:** 2024-07-18

In the Original Investigation titled “Enhanced Recovery After Surgery Guidelines and Hospital Length of Stay, Readmission, Complications, and Mortality: A Meta-Analysis of Randomized Clinical Trials,”^1^ published June 18, 2024, Figure 2 contained a numerical error in the upper bound of the data point for bariatric surgery, and the x-axis was mislabeled as the risk ratio instead of the mean difference. There were also spelling errors in Figures 1 and 3. This article has been corrected.^1^
