# Supplementary material for: Enhanced Recovery After Surgery Guidelines and Hospital Length of Stay, Readmission, Complications, and Mortality: A Meta-Analysis of Randomized Clinical Trials
Source: JAMA Netw Open. 2024 Jun 18;7(6):e2417310. doi: 10.1001/jamanetworkopen.2024.17310 (PMC11195621; doi:10.1001/jamanetworkopen.2024.17310)
Supplement: Supplement 1. — eAppendix 1. Search Strategy eAppendix 2. Data Dictionary eTable 1. Characteristics of Included Studies eTable 2. Risk of Bias eFigure. PRISMA Flow Diagram [file jamanetwopen-e2417310-s001.pdf]

Supplemental Online Content

Sauro KM, Smith C, Ibadin S, et al. Enhanced Recovery After Surgery guidelines and hospital length of stay, readmission, complications, and mortality. *JAMA Netw Open.* 2024;7(6):e2417310. doi:10.1001/jamanetworkopen.2024.17310

eAppendix 1. Search Strategy

eAppendix 2. Data Dictionary

eTable 1. Characteristics of Included Studies

eTable 2. Risk of Bias

eFigure. PRISMA Flow Diagram

This supplemental material has been provided by the authors to give readers additional information about their work.

© 2024 Sauro KM et al. *JAMA Network Open*.

eAppendix 1. Search strategies

Database(s): **Ovid MEDLINE(R) and Epub Ahead of Print, In-Process, In-Data-Review & Other Non-Indexed Citations and Daily** 1946 to June 18, 2021
Search Strategy:

#	Searches	Results
1	exp Enhanced Recovery After Surgery/	659
2	(ERAS or ERP or "enhanced recovery" or "enhanced rehabilitation" or "fast track" or FTS).kf,tw.	29740
3	1 or 2	29794
4	Practice Guidelines as Topic/	123943
5	Critical Pathways/	7164
6	(guideline* or pathway* or protocol*).kf,tw.	2041178
7	4 or 5 or 6	2105525
8	3 and 7	4996

Database(s): **Embase** 1974 to 2021 June 21
Search Strategy:

#	Searches	Results
1	enhanced recovery after surgery/	1809
2	(ERAS or ERP or "enhanced recovery" or "enhanced rehabilitation" or "fast track" or FTS).kw,tw.	42713
3	1 or 2	42975
4	practice guideline/ or clinical pathway/	471208
5	(guideline* or pathway* or protocol*).kw,tw.	2790188
6	4 or 5	2995615
7	3 and 6	9010
8	limit 7 to (editorial or letter or note)	250
9	7 not 8	8760

Database(s): **EBM Reviews - Cochrane Central Register of Controlled Trials** May 2021
Search Strategy:

#	Searches	Results
1	(ERAS or ERP or "enhanced recovery" or "enhanced rehabilitation" or "fast track" or FTS).kw,tw.	4013
2	Practice Guidelines as Topic/	1675

© 2024 Sauro KM et al. *JAMA Network Open.*

3	Critical Pathways/	203
4	(guideline* or pathway* or protocol*).kw,tw.	172695
5	2 or 3 or 4	173219
6	1 and 5	1275

Database(s): **EBM Reviews - Cochrane Central Register of Controlled Trials** May 2021
Search Strategy:

#	Searches	Results
1	(ERAS or ERP or "enhanced recovery" or "enhanced rehabilitation" or "fast track" or FTS).kw,tw.	4013
2	Practice Guidelines as Topic/	1675
3	Critical Pathways/	203
4	(guideline* or pathway* or protocol*).kw,tw.	172695
5	2 or 3 or 4	173219
6	1 and 5	1275

*Note: There were no articles indexed with the SH "Enhanced Recovery After Surgery" which is why it's not present in the search history.

© 2024 Sauro KM et al. *JAMA Network Open.*

eAppendix 2. Data dictionary

Variable name	Description	Possible values
**Master_ERAS Data**		
Country		Open text
Province/State, City, Region	Provide as much information provided in manuscript regarding geographical location	Open text
Setting	Information about size and location of hospital	Open text
Hospital type(s)	Description of type of hospital (e.g. tertiary, teaching, trauma, etc.)	Open text
Hospital number	Number of included hospitals	N
Bed numbers	Number of beds in hospital	N
Start year data collection	Year data collection began	Year
End year data collection	Year data collection ended	Year
Study design	Epidemiological study design<br>*this will determine quality assessment criteria	Cohort<br>Case control<br>RCT<br>Intervention<br>Quasi-experimental<br>Other
Timing of data collection	Timing related to patient admission	Prospective<br>Retrospective<br>Cross-sectional
Data collection method	How was ERAS Ascertained	Administrative data<br>Chart review<br>Voluntary reporting<br>Other
Reviewer process	If ERAS ascertainment by chart review, provide information about reviewer	Number of reviewers, role (e.g. nurse, MD, research staff, other)
Exclusion criteria	Description of any exclusion criteria (except pediatric)	Open text

© 2024 Sauro KM et al. *JAMA Network Open.*

Patient number	Number of included patients	N
Non-ERAS, before group	Number of patients in Non-ERAS group	N
ERAS, after group	Number of patients in ERAS group	N
Age describe	Describe age of population	Yes<br>No
Age (type)	Description of how age is presented	Range<br>Mean<br>Median<br>Categories
Age (range)	Provide overall age range	Years of age
Age (overall, mean)	Provide overall mean	Years of age
Age (overall, median)	Provide overall median	Years of age
Age (ERAS, mean)	Provide mean age of ERAS group	Years of age
Age (non-ERAS, mean)	Provide mean age of non-ERAS group	Years of age
Age (ERAS, median)	Provide mean age of ERAS group	Years of age
Age (non-ERAS, median)	Provide mean age of non-ERAS group	Years of age
Sex (male)	Provide proportion of population/sample male	Proportion
Surgery Category	Surgery Category (ERAS)	Gynecological<br>Colorectal<br>Liver<br>Pancreas<br>Breast<br>Bariatric<br>Head & Neck<br>Orthopedic<br>Other -Specify
Surgery Describe	Description of Surgery Types Included	Open text
ERAS Protocol Label	Provide how the authors describe the surgical protocol (ERAS/ERP/Fast-Track etc)	Open Text
ERAS 1 Preadmission Patient Education	Is ERAS element included in the study's surgery protocol	Yes<br>No<br>NR (not reported)

© 2024 Sauro KM et al. *JAMA Network Open.*

ERAS 2 Preadmission Screening (nutrition, frailty, tobacco, ethanol)	Is ERAS element included in the study's surgery protocol	Yes<br>No<br>NR (not reported)
ERAS 3 Fasting and Carbohydrate Loading	Is ERAS element included in the study's surgery protocol	Yes<br>No<br>NR (not reported)
ERAS 4 Pre-emptive Analgesia	Is ERAS element included in the study's surgery protocol	Yes<br>No<br>NR (not reported)
ERAS 5 Anti-emetic Prophylaxis	Is ERAS element included in the study's surgery protocol	Yes<br>No<br>NR (not reported)
ERAS 6 Intraoperative Fluid Management Strategy	Is ERAS element included in the study's surgery protocol	Yes<br>No<br>NR (not reported)
ERAS 7 Anesthetics Administered (dose, route, timing)	Is ERAS element included in the study's surgery protocol	Yes<br>No<br>NR (not reported)
ERAS 8 Patient Warming	Is ERAS element included in the study's surgery protocol	Yes<br>No<br>NR (not reported)
ERAS 9 Management of Postoperative Fluids	Is ERAS element included in the study's surgery protocol	Yes<br>No<br>NR (not reported)
ERAS 10 Postoperative Analgesia and Anti-emetic plans	Is ERAS element included in the study's surgery protocol	Yes<br>No<br>NR (not reported)
ERAS 11 Plan for Opioid Minimization	Is ERAS element included in the study's surgery protocol	Yes<br>No<br>NR (not reported)
ERAS 12 Drain and Line Management	Is ERAS element included in the study's surgery protocol	Yes<br>No<br>NR (not reported)
ERAS 13 Early Mobilization	Is ERAS element included in the study's surgery protocol	Yes<br>No<br>NR (not reported)
ERAS 14 Postoperative Diet and Bowel Regimen Management	Is ERAS element included in the study's surgery protocol	Yes<br>No<br>NR (not reported)
ERAS 15 Criteria for Discharge	Is ERAS element included in the study's surgery protocol	Yes<br>No<br>NR (not reported)
ERAS 16 Tracking of Post Discharge Outcomes		Yes<br>No<br>NR (not reported)

© 2024 Sauro KM et al. *JAMA Network Open.*

ERAS Criteria Notes	Optional: notes on ERAS elements and criteria	Open Text
ERAS Compliance Describe	Provide compliance estimate for ERAS	Yes<br>No
ERAS Compliance Estimate	Provide compliance estimate for ERAS	Yes<br>No
Implementation Interventions Describe	Description of any implementation interventions noted in the text.	Open Text
**LOS WORKSHEET**		
Overall estimate	Is this an overall estimate	Yes<br>No
Stratification	Were stratified estimates provided?<br>*should be "No" if answer to overall is "Yes"	Yes<br>No
Sex strat	Sex stratified estimate	Yes<br>No
Sex	What sex is estimate provided for	Male<br>Female
Age strat	Age stratified estimate	Yes<br>No
Age group	What sex is estimate provided for	Age category
Year strat	Year stratified estimate	Yes<br>No
Year range	Year range for strata (e.g. 2001-2002)	Years
Other strat	Estimate stratified by other variable	Yes<br>No
Other strat, specify	Name of variable stratified	Open text
Other category	Category estimate stratified by	Open text
Control_Estimate	Provide Length of Stay	Days
Control_N	Provide N of control (non-ERAS) group	N
Control_SD	Provide Standard Deviation of Estimate	N
Lower 95% CI/ IQR/ Range	Provide lower 95%CI (IQR or Range if Median estimate)	N
Upper 95% CI/ IQR/ Range	Provide upper 95%CI (IQR or Range if Median estimate)	N

© 2024 Sauro KM et al. *JAMA Network Open.*

ERAS_Estimate	Provide Length of Stay	Days
ERAS_N	Provide N of ERAS group	N
ERAS_SD	Provide Standard Deviation of Estimate	N
Lower 95% CI/ IQR/ Range	Provide lower 95%CI (IQR or Range if Median estimate)	N
Upper 95% CI/ IQR/ Range	Provide upper 95%CI (IQR or Range if Median estimate)	N
Estimate_type		Mean<br>Median<br>Patient
Estimate_unit	Provide the estimate unite	Days
Estimate_describe	Describe the estimate	
**Complications Worksheet**		
Postoperative Complications Describe	Describe postoperative complications	Open text
Postoperative Complications Estimate	Provide estimate for postoperative complications	N per 100
Overall estimate	Is this an overall estimate	Yes<br>No
Stratification	Were stratified estimates provided?<br>*should be “No” if answer to overall is “Yes”	Yes<br>No
Sex strat	Sex stratified estimate	Yes<br>No
Sex	What sex is estimate provided for	Male<br>Female
Age strat	Age stratified estimate	Yes<br>No
Age group	What sex is estimate provided for	Age category
Year strat	Year stratified estimate	Yes<br>No
Year range	Year range for strata (e.g. 2001-2002)	Years
Other strat	Estimate stratified by other variable	Yes<br>No
Other strat, specify	Name of variable stratified	Open text (ex. ae type, severity)
Other category	Category estimate stratified by	Open text

© 2024 Sauro KM et al. *JAMA Network Open.*

Control_Estimate	Provide # of complications in control group	N
Control_N	Provide N of control (non-ERAS) group	N
ERAS_Estimate	Provide # of complications in ERAS group	N
ERAS_N	Provide N of ERAS group	N
Unit	Provide Estimate Unit	Patient<br>N
Estimate_Describe	Describe the Estimate	Open Text
**Mortality Worksheet**		
Overall estimate	Is this an overall estimate	Yes<br>No
Stratification	Were stratified estimates provided?<br>*should be "No" if answer to overall is "Yes"	Yes<br>No
Sex strat	Sex stratified estimate	Yes<br>No
Sex	What sex is estimate provided for	Male<br>Female
Age strat	Age stratified estimate	Yes<br>No
Age group	What sex is estimate provided for	Age category
Year strat	Year stratified estimate	Yes<br>No
Year range	Year range for strata (e.g. 2001-2002)	Years
Other strat	Estimate stratified by other variable	Yes<br>No
Other strat, specify	Name of variable stratified	Open text
Other category	Category estimate stratified by	Open text
Control_Estimate	Provide # of deaths in Control Group	N
Control_N	Provide N of control (non-ERAS) group	N
ERAS_Estimate	Provide # of deaths in ERAS group	N
ERAS_N	Provide N of ERAS group	N
Unit	Provide Estimate Unit	Patient
Estimate_Describe	Describe the Estimate	Open Text

© 2024 Sauro KM et al. *JAMA Network Open.*

**Readmission Worksheet**		
Overall estimate	Is this an overall estimate	Yes<br>No
Stratification	Were stratified estimates provided?<br>*should be “No” if answer to overall is “Yes”	Yes<br>No
Sex strat	Sex stratified estimate	Yes<br>No
Sex	What sex is estimate provided for	Male<br>Female
Age strat	Age stratified estimate	Yes<br>No
Age group	What sex is estimate provided for	Age category
Year strat	Year stratified estimate	Yes<br>No
Year range	Year range for strata (e.g. 2001-2002)	Years
Other strat	Estimate stratified by other variable	Yes<br>No
Other strat, specify	Name of variable stratified	Open text
Other category	Category estimate stratified by	Open text
Control_Estimate	Provide # of readmissions in Control Group	N
Control_n	Provide N of control (non-ERAS) group	N
ERAS_Estimate	Provide # of readmissions in ERAS group	N
ERAS_n	Provide N of ERAS group	N
Estimate_Unit	Provide Estimate Unit	Patient<br>n
Estimate_Describe	Describe the Estimate	Open Text
**Cost Worksheet**		
Overall estimate	Is this an overall estimate	Yes<br>No
Stratification	Were stratified estimates provided?<br>*should be “No” if answer to overall is “Yes”	Yes<br>No
Sex strat	Sex stratified estimate	Yes<br>No

© 2024 Sauro KM et al. *JAMA Network Open.*

Sex	What sex is estimate provided for	Male<br>Female
Age strat	Age stratified estimate	Yes<br>No
Age group	What sex is estimate provided for	Age category
Year strat	Year stratified estimate	Yes<br>No
Year range	Year range for strata (e.g. 2001-2002)	Years
Other strat	Estimate stratified by other variable	Yes<br>No
Other strat, specify	Name of variable stratified	Open text
Other category	Category estimate stratified by	Open text
Control_Estimate	Provide cost estimate in Control Group	N
Control_SD	Provide N of control (non-ERAS) group	N
Lower 95% CI/ IQR/ Range	Provide lower 95%CI (IQR or Range if Median estimate)	N
Upper 95% CI/ IQR/ Range	Provide upper 95%CI (IQR or Range if Median estimate)	N
ERAS_Estimate	Provide cost estimate in ERAS group	N
ERAS_SD	Provide N of ERAS group	N
Lower 95% CI/ IQR/ Range	Provide lower 95%CI (IQR or Range if Median estimate)	N
Upper 95% CI/ IQR/ Range	Provide upper 95%CI (IQR or Range if Median estimate)	N
Estimate Difference	ERAS estimate – Control Estimate	N
Unit	Provide Estimate Unit	CAD<br>USD<br>Euro<br>Etc.
Per Unit	Provide the Per Unit	Per patient<br>Per procedure<br>Per hospital
Estimate_Type	Provide the estimate type	Mean<br>Median

© 2024 Sauro KM et al. *JAMA Network Open.*

Estimate_Describe	Describe the Estimate	Open Text
**Quality Assessment**		
RECOVER CHECKLIST	See QA data dictionary	
NOS	See QA data dictionary	
ROB-2	See QA data dicitonary	

© 2024 Sauro KM et al. *JAMA Network Open.*

eTable 1. Characteristics of included studies

Author	Year	Country	Surgery Category	Study Design	Outcomes	N Control ERAS	Protocol Type	#ERAS Elements	Risk of Bias	Recover
**AbdElRahman**	2020	Egypt	Colorectal	RCT	Hospital LoS<br>Complications<br>Hospital readmit	80<br>40<br>40	ERAS	12	Moderate	12
**Abdikarim**	2015	China	Upper GI	RCT	Hospital LoS<br>Complications<br>Hospital readmit<br>Mortality	61<br>30<br>31	ERAS	11	Moderate	9
**Akca**	2019	Turkey	Gynecological	RCT	Hospital LoS<br>Hospital readmit	89<br>45<br>44	ERAS	14	Moderate	15
**Akhtar**	2020	Pakistan	Other	RCT	Hospital LoS<br>Complications<br>Hospital readmit<br>Cost	147<br>74<br>73	ERAS	11	Moderate	13
**Baluku**	2020	Uganda	Gynaecologic	RCT	Hospital LoS<br>Complications<br>Hospital readmit	160<br>80<br>80	ERAS	12	High	16
**Bansal**	2020	India	Urology	RCT	Hospital LoS<br>Complications<br>Hospital readmit<br>Mortality	54<br>27<br>27	ERAS	8	Low	15
**Chndan**	2020	India	Upper GI	RCT	Hospital LoS<br>Complications	85<br>43<br>42	ERAS	6	Moderate	11
**Cui**	2019	China	Orthopedic	RCT	Complications	50<br>25<br>25	ERAS	6	Moderate	14

© 2024 Sauro KM et al. *JAMA Network Open.*

Author	Year	Country	Surgery Category	Study Design	Outcomes	N<br>Control<br>ERAS	Protocol Type	#ERAS Elements	Risk of Bias	Recover
**Damadi**	2019	USA	Colorectal	RCT	Hospital LoS<br>Hospital readmit	123<br>41<br>51	ERP	3	Low	13
**Dambaev**	2020	Russia	Other	RCT	Hospital LoS<br>Complications<br>Mortality	174<br>87<br>87	ERAS	14	Moderate	11
**Dickson**	2017	USA	Gynaecologic	RCT	Hospital LoS<br>Complications<br>Hospital readmit	103<br>52<br>51	ERAS Protocol	11	Moderate	13
**Fei**	2015	China	Upper GI	RCT	Hospital LoS<br>Complications<br>Hospital readmit<br>Mortality	116<br>57<br>59	Fast-track Surgery	9	Moderate	13
**Feng**	2014	China	Colorectal	RCT	Complications	116<br>59<br>57	Fast Track	6	Low	12
**Feo**	2016	Italy	Other	Cohort	Hospital LoS<br>Complications<br>Hospital readmit<br>Mortality	121<br>91<br>130	ERP	12	Low	16
**Ferrari**	2020	Italy	Gynaecologic	RCT	Hospital LoS<br>Complications<br>Hospital readmit	168<br>85<br>83	ERAS	14	Moderate	18
**Forsmo**	2016	Norway	Colorectal	RCT	Hospital LoS<br>Complications<br>Hospital readmit<br>Mortality	183<br>122<br>61	ERAS	12	Low	14

© 2024 Sauro KM et al. *JAMA Network Open.*

Author	Year	Country	Surgery Category	Study Design	Outcomes	N Control ERAS	Protocol Type	#ERAS Elements	Risk of Bias	Recover
**Forsmo**	2016	Norway	Colorectal	RCT	Hospital LoS<br>Complications<br>Hospital readmit<br>Mortality	324<br>162<br>162	ERAS	15	Moderate	16
**Han**	2019	China	Other	RCT	Hospital LoS<br>Hospital readmit<br>Mortality	300<br>150<br>150	ERAS protocol	13	Moderate	11
**He**	2015	China	Upper GI	RCT	Hospital LoS<br>Complications<br>Hospital readmit<br>Mortality	86<br>38<br>48	ERP	13	Moderate	15
**Hwang**	2019	Korea	Other	RCT	Hospital LoS<br>Complications<br>Hospital readmit<br>Mortality<br>Cost	247<br>124<br>123	ERAS	12	Low	16
**Ibrahim**	2020	Egypt	Colorectal	RCT	Hospital LoS<br>Complications<br>Mortality	75<br>43<br>32	ERAS Protocol	8	Moderate	9
**Ionescu**	2009	Romania	Colorectal	RCT	Complications	96<br>48<br>48	Fast Track	8	Moderate	12
**Iyer**	2019	India	Upper GI	RCT	Hospital LoS	100<br>50<br>50	Fast-track	8	High	10
**Jones**	2021	UK	Liver	RCT	Hospital LoS<br>Mortality<br>Cost	91<br>45<br>46	ERP	15	Low	14

© 2024 Sauro KM et al. *JAMA Network Open.*

Author	Year	Country	Surgery Category	Study Design	Outcomes	N Control ERAS	Protocol Type	#ERAS Elements	Risk of Bias	Recover
**Kang**	2018	Korea	Other	RCT	Hospital LoS<br>Complications<br>Hospital readmit<br>Mortality	97<br>51<br>46	ERAS Protocol	14	Moderate	15
**Kapritsou**	2020	Greek/Greece	Pancreatic	RCT	Hospital LoS<br>Complications	85<br>41<br>44	ERAS Protocol	7	Moderate	15
**Kapritsou**	2018	Greek/Greece	Liver	RCT	Hospital LoS<br>Complications<br>Hospital readmit	62<br>30<br>32	Fast-Track Recovery Program	11	High	9
**Kilpio**	2020	Finland	Gynaecologic	RCT	Hospital LoS<br>Complications<br>Hospital readmit	120<br>60<br>60	ERAS Protocol	15	Moderate	14
**Lavu**	2019	USA	Pancreas	RCT	Hospital LoS<br>Complications<br>Hospital readmit<br>MortalityCost	76<br>39<br>37	Whipple Accelerated Recovery Pathway (WARP)	6	Low	14
**Lemanu**	2013	New Zealand	Bariatric	RCT	Hospital LoS<br>Complications<br>Hospital readmit<br>Cost	121<br>38<br>40	Enhanced recovery	11	Moderate	17
**Li**	2018	China	Cardiac	RCT	Hospital LoS<br>Complications<br>Hospital readmit<br>Mortality<br>Cost	209<br>105<br>104	ERAS	12	Low	16
**Liang**	2018	China	Liver	RCT	Hospital LoS<br>Complications<br>Hospital readmit<br>Mortality Cost	119<br>61<br>58	ERAS	16	Low	17

© 2024 Sauro KM et al. *JAMA Network Open.*

Author	Year	Country	Surgery Category	Study Design	Outcomes	N Control ERAS	Protocol Type	#ERAS Elements	Risk of Bias	Recover
**Liu**	2019	China	Other	RCT	Hospital readmit<br>Mortality	140<br>70<br>70	ERAS	14	Moderate	15
**Liu**	2020	China	Orthopedic	RCT	Hospital LoS<br>Complications<br>Hospital readmit<br>Mortality<br>Cost	94<br>46<br>48	ERAS	14	Low	15
**Liu**	2016	China	Upper GI	RCT	Hospital LoS<br>Complications<br>Cost	42<br>21<br>21	Fast Track	8	Moderate	12
**Mari**	2016	Italy	Colorectal	RCT	Hospital LoS<br>Complications<br>Hospital readmit	83<br>43<br>40	ERAS	7	Moderate	12
**Mari**	2014	Italy	Colorectal	RCT	Hospital LoS<br>Complications<br>Hospital readmit	50<br>25<br>25	Fast Track	9	High	9
**Mingjie**	2015	China	Other	RCT	Hospital LoS<br>Complications<br>Hospital readmit<br>Mortality	149<br>73<br>76	ERAS	13	Moderate	14
**Mohsina**	2018	India	Other	RCT	Hospital LoS<br>Complications	99<br>49<br>50	ERAS Pathway	11	Low	14
**Muller**	2009	Switzerlan d	Colorectal	RCT	Complications<br>Hospital readmit<br>Mortality	151<br>75<br>76	adapted fast-track protocol	7	Moderate	15

© 2024 Sauro KM et al. *JAMA Network Open.*

Author	Year	Country	Surgery Category	Study Design	Outcomes	N Control ERAS	Protocol Type	#ERAS Elements	Risk of Bias	Recover
**Nakanishi**	2020	Japan	Liver	RCT	Hospital LoS<br>Complications<br>Mortality	57<br>28<br>29	ERAS	10	High	15
**Nunez-Venzor**	2021	Mexico	Upper GI	RCT	Hospital LoS<br>Complications<br>Hospital readmit	38<br>19<br>19	ERAS Protocol	14	High	14
**Ostermann**	2019	Switzerland	Colorectal	RCT	Hospital LoS<br>Complications<br>Hospital readmit	150<br>75<br>75	Enhanced Recovery Program	13	Low	18
**Pan**	2020	China	Other	RCT	Hospital LoS<br>Complications<br>Cost	216<br>104<br>112	ERAS Protocol	14	Moderate	15
**Peng**	2021	China	Gynaecologic	RCT	Hospital LoS<br>Complications<br>Hospital readmit<br>Mortality<br>Cost	130<br>65<br>65	ERAS	12	Moderate	13
**Pirzada**	2017	Pakistan	Upper GI	RCT	Hospital LoS<br>Complications	60<br>30<br>30	ERAS protocol	13	High	10
**Prabhakaran**	2020	India	Bariatric	RCT	Hospital LoS<br>Complications<br>Hospital readmit	112<br>56<br>56	ERAS Pathway	13	Moderate	13
**Qu**	2020	China	Other	RCT	Hospital LoS<br>Complications<br>Cost<br>Hospital readmit	129<br>65<br>64	ERAS Protocol	15	Moderate	14
**Rao**	2017	China	Liver	RCT	Hospital LoS<br>Complications<br>Hospital readmit<br>Mortality	128<br>74<br>54	Fast-track	13	High	12

© 2024 Sauro KM et al. *JAMA Network Open.*

Author	Year	Country	Surgery Category	Study Design	Outcomes	N<br>Control<br>ERAS	Protocol Type	#ERAS Elements	Risk of Bias	Recover
**Ren**	2012	China	Colorectal	RCT	Hospital LoS<br>Complications<br>Mortality<br>Cost	597<br>298<br>299	ERAS	13	High	14
**Ruiz-Tovar**	2019	Spain	Bariatric	RCT	Hospital LoS<br>Complications<br>Hospital readmit<br>Mortality	180<br>90<br>90	ERAS	15	Moderate	12
**Sanchez-Iglesias**	2020	Spain	Gynaecologic	RCT	Hospital LoS<br>Complications<br>Hospital readmit<br>Mortality	99<br>49<br>50	ERAS	16	High	17
**Sanchez-Iglesias**	2021	Spain	Gynaecologic	RCT	Hospital LoS	99<br>49<br>50	ERAS	16	Low	15
**Saurabh**	2020	India	Other	RCT	Hospital LoS<br>Complications<br>Hospital readmit<br>Mortality	70<br>35<br>35	ERAS	11	High	15
**Sazhin**	2018	Russia	Upper GI	RCT	Hospital LoS<br>Complications<br>Hospital readmit<br>Mortality	86<br>48<br>38	Fast Track	12	High	10
**Sazhin**	2018	Russia	Upper GI	RCT	Hospital LoS<br>Complications<br>Hospital readmit<br>Mortality	102<br>57<br>45	Fast-track	11	High	10
**Scioscia**	2017	Italy	Colorectal	RCT	Hospital LoS<br>Complications<br>Hospital readmit<br>Cost	277<br>165<br>62	Fast-track	5	Moderate	13

© 2024 Sauro KM et al. *JAMA Network Open.*

Author	Year	Country	Surgery Category	Study Design	Outcomes	N Control ERAS	Protocol Type	#ERAS Elements	Risk of Bias	Recover
**Serclova**	2009	Czech Republic	Colorectal	RCT	Hospital LoS<br>Complications<br>Hospital readmit<br>Mortality	103<br>52<br>51	Fast-track Protocol	11	High	11
**Serclova**	5943	Czech Republic	Colorectal	RCT	Hospital LoS<br>Complications<br>Hospital readmit<br>Mortality	103<br>52<br>51	Fast-track Protocol	12	High	10
**Sharma**	2021	India	Upper GI	RCT	Hospital LoS<br>Complications<br>Hospital readmit<br>Mortality	100<br>50<br>50	ERAS Protocol	12	Moderate	13
**Shetiwy**	2017	Egypt	Colorectal	RCT	Hospital LoS<br>Complications<br>Hospital readmit<br>Mortality	70<br>35<br>35	ERAS	14	Moderate	12
**Soffin**	2020	USA	Orthopedic	RCT	Complications	51<br>25<br>26	ERAS	10	Low	17
**Swaminathan**	2020	India	Upper GI	RCT	Hospital LoS<br>Complications	58<br>29<br>29	ERAS	13	Moderate	14
**Takagi**	2019	Japan	Pancreas	RCT	Hospital LoS<br>Complications<br>Hospital readmit<br>Mortality<br>Cost	74<br>37<br>37	ERAS	13	Moderate	18
**Tanaka**	2017	Japan	Upper GI	RCT	Hospital LoS<br>Complications<br>Hospital readmit<br>Cost	142<br>69<br>73	ERAS protocol	11	Low	15

© 2024 Sauro KM et al. *JAMA Network Open.*

Author	Year	Country	Surgery Category	Study Design	Outcomes	N Control ERAS	Protocol Type	#ERAS Elements	Risk of Bias	Recover
**Taupyk**	2015	China	Colorectal	RCT	Hospital LoS<br>Complications	70<br>39<br>31	Fast-track	8	High	9
**Teigen**	2020	USA	Gynecology	RCT	Hospital LoS<br>Complications<br>Hospital readmit	118<br>60<br>58	ERAS	7	Moderate	15
**Trejo-Avila**	2019	Mexico	Upper GI	RCT	Hospital LoS<br>Complications<br>Hospital readmit	108<br>58<br>50	ERAS	9	Moderate	14
**Vlad**	2020	Romania	Bladder Cancer	RCT	Hospital LoS<br>Complications<br>Hospital readmit<br>Mortality	90<br>45<br>45	ERAS	11	Moderate	15
**Wang**	2019	China	Orthopedic	RCT	Hospital LoS<br>Complications<br>Hospital readmit<br>MortalityCost	140<br>70<br>70	ERAS	10	Moderate	15
**Yang**	2016	China	orthopedic	RCT	Hospital LoS<br>Complications<br>Hospital readmit<br>Mortality	258<br>126<br>132	Fast Track	11	Moderate	15
**Yilmaz**	2020	Turkey	Gynecological	RCT	Hospital LoS<br>Complications<br>Hospital readmit	104<br>53<br>51	ERAS	10	High	14
**Yilmaz**	2018	Turkey	Gynecological	RCT	Hospital LoS<br>Complications<br>Hospital readmit	62<br>32<br>30	ERAS	14	High	14
**Zhao**	2014	China	Upper GI	RCT	Hospital LoS<br>Complications<br>Hospital readmit<br>Cost	68<br>34<br>34	Fast Track	8	High	12

© 2024 Sauro KM et al. *JAMA Network Open.*

eTable 2. Risk of bias

Randomization process	Deviation from intended intervention (intent to treat)	Deviation from intended intervention (per protocol)	Missing Outcome data	Measurement of Outcome	Selection of results
Low = 52.0 (39) Some = 42.7 (32) High = 5.3 (4)	Low = 44.0 (33) Some = 40.0 (30) High = 16 (12)	Low = 52.0 (39) Some = 22.7 (17) High = 22.7 (17)	Low = 85.3 (64) Some = 8.0 (6) High = 5.4 (4)	Low = 65.3 (49) Some = 26.7 (20) High = 8.0 (6)	Low = 76.0 (57) Some = 24.0 (18) High = 0

© 2024 Sauro KM et al. *JAMA Network Open*.

eFigure. PRISMA Flow Diagram

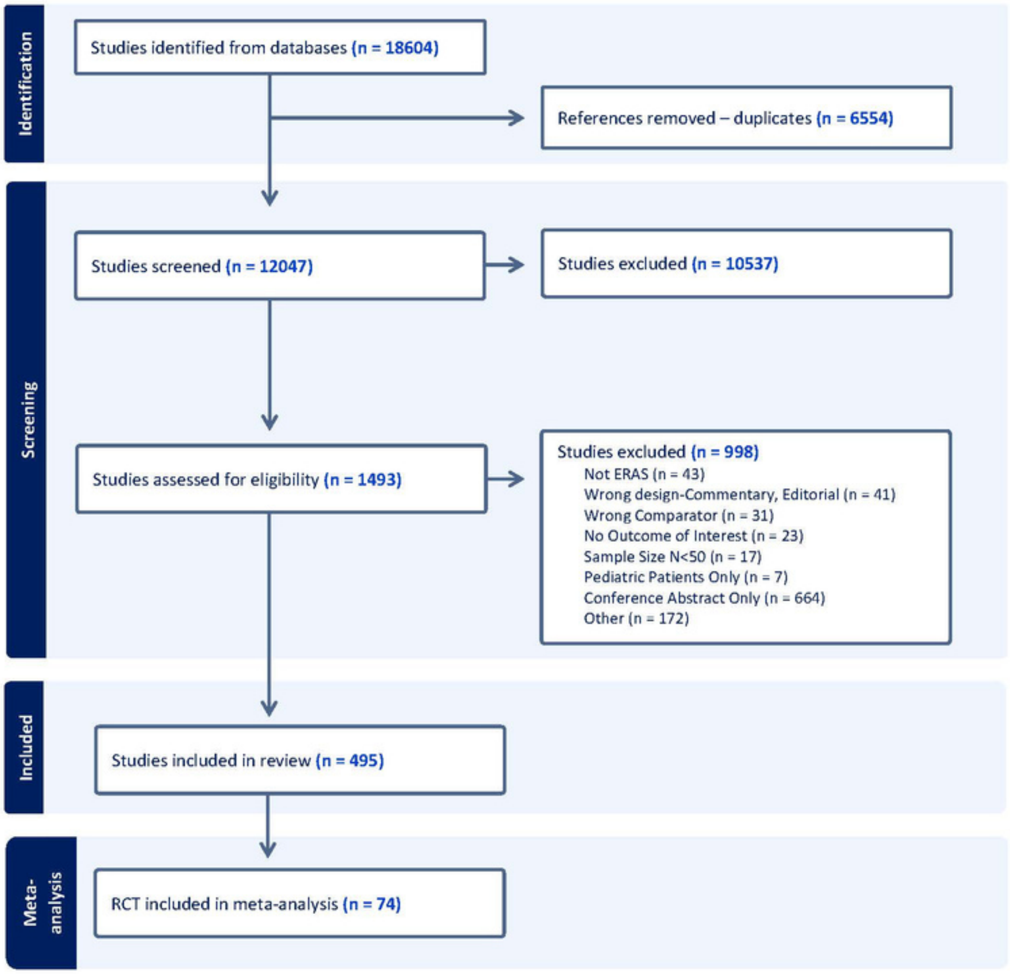

© 2024 Sauro KM et al. *JAMA Network Open.*
