# Supplementary material for: Enhanced Recovery After Surgery Guidelines and Hospital Length of Stay, Readmission, Complications, and Mortality: A Meta-Analysis of Randomized Clinical Trials
Source: JAMA Netw Open. 2024 Jun 18;7(6):e2417310. doi: 10.1001/jamanetworkopen.2024.17310 (PMC11195621; doi:10.1001/jamanetworkopen.2024.17310)
Supplement: Supplement 2. — Data Sharing Statement [file jamanetwopen-e2417310-s002.pdf]

Data Sharing Statement

Sauro. Enhanced Recovery After Surgery Guidelines and Hospital Length of Stay, Readmission, Complications, and Mortality. *JAMA Netw Open*. Published June 17, 2024. doi:10.1001/jamanetworkopen.2024.17310

Data
Data available: Yes
Data types: Data (not involving human participants)
How to access data: kmsauro@ucalgary.ca
When available: With publication

Supporting Documents
Document types: Statistical/analytic code
How to access documents: kmsauro@ucalgary.ca
When available: With publication

Additional Information
Who can access the data: Data will be provided upon reasonable request to the corresponding author
Types of analyses: All analyses
Mechanisms of data availability: Data will be provided upon reasonable request to the corresponding author
